# Anti-Ri Paraneoplastic Neurologic Syndrome in a Patient With Untreated Breast Cancer

**DOI:** 10.7759/cureus.91743

**Published:** 2025-09-06

**Authors:** David X Li, Tyler Jarrett, Jessica Ma, Jonathan F Garcia

**Affiliations:** 1 Internal Medicine, University of California Los Angeles, Los Angeles, USA; 2 Hospital Medicine, University of California Los Angeles, Los Angeles, USA

**Keywords:** anti-ri antibody, breast cancer, opsoclonus-myoclonus syndrome, paraneoplastic, paraneoplastic syndrome

## Abstract

Paraneoplastic syndromes (PNS) refer to a broad range of disorders that can impact any part of the nervous system and are frequently immune-mediated. Although classically associated with solid tumor malignancies, PNS remain a rare phenomenon. Anti-Ri-positive (antineuronal nuclear antibody type 2-positive) PNS is highly associated with breast cancer and results in a variety of clinical presentations, including cerebellar degeneration and opsoclonus-myoclonus syndrome. In this report, we describe a case of anti-Ri-positive PNS identified in a patient with a several-year history of untreated localized breast malignancy.

## Introduction

Breast cancer is the most commonly diagnosed cancer worldwide; in the United States, approximately 300,000 new cases are diagnosed annually [1]. Paraneoplastic neurologic syndromes (PNS) related to breast cancer, on the other hand, are rare, with some studies suggesting an estimated incidence of less than one percent for most solid tumors [2]. PNS refer to a broad range of disorders that can impact any part of the nervous system and are frequently immune-mediated. In breast cancer, presentations including sensory- and motor-type neuropathies, cerebellar degeneration, stiff person syndrome, and opsoclonus-myoclonus syndrome have been described [3]. Due to the variation in clinical presentations and wide differential diagnoses, PNS are challenging to diagnose. Antibody testing is frequently utilized to aid in the identification of PNS and their associated malignancies, although certain antibodies are classified as high risk (that is, in cases with detectable antibodies, greater than 70% are associated with malignancy), while others are much less specific for malignancy [4].

In this report, we describe a case of anti-Ri-positive (antineuronal nuclear antibody type 2-positive) PNS identified in a patient with a several-year history of untreated, localized breast malignancy.

## Case presentation

A 60-year-old female with a history of untreated invasive ductal carcinoma of the right breast presented to the emergency room with eight months of progressively worsening generalized weakness. She had been diagnosed with a right breast mass four years prior, with pathology demonstrating invasive ductal carcinoma with positive expression of estrogen receptors (ER) and progesterone receptors (PR) and negative expression of human epidermal growth factor receptor 2 (HER-2). Following the initial diagnosis, she sought alternative treatments and was lost to medical follow-up. In the eight months prior to the current presentation, she had experienced progressively worsening bilateral upper and lower extremity weakness and tremors, resulting in significant debility.

**Figure 1:**
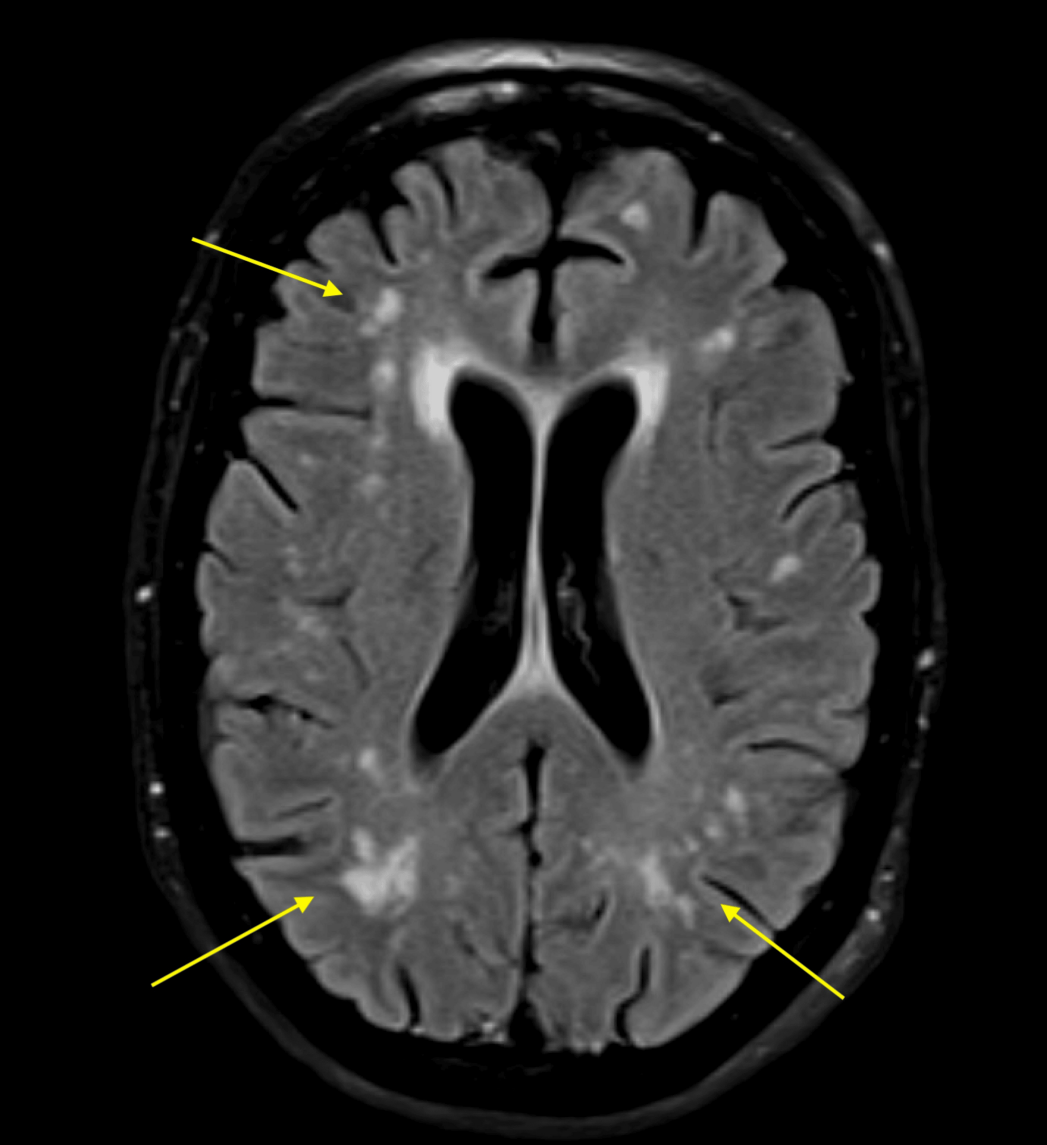
Magnetic resonance imaging of the brain demonstrating patchy supratentorial white matter hyperintensities on fluid attenuated inversion recovery (FLAIR) images

On examination, there was weakness and contractures of the bilateral upper and lower extremities, severe intention tremor, and dysarthria; sensation was intact. Mild hyperreflexia was noted in the bilateral lower extremities. Laboratory evaluation was notable for a white blood cell count of 11.84 x10E3/uL; otherwise, a complete blood count and metabolic panel did not demonstrate abnormal findings (Table 1). Computed tomography of the chest, abdomen, and pelvis demonstrated a lobulated mass in the right breast without evidence of metastatic disease. Magnetic resonance imaging of the brain and spine demonstrated moderate patchy supratentorial white matter hyperintensities on fluid attenuated inversion recovery (FLAIR) images (Figure 1). Lumbar puncture was performed; cerebrospinal fluid (CSF) analysis was unremarkable, aside from a mildly elevated protein of 55 mg/dL (Table 2), and CSF bacterial, fungal, and acid-fast cultures were negative. The CSF paraneoplastic panel demonstrated a positive anti-Ri autoantibody assay. A diagnosis of paraneoplastic syndrome was made. 

**Table 1:** Serum laboratory evaluation upon hospital admission

Test	Value	Reference range
Complete blood count		
White blood cell count	11.84 ×10³/µL	4.0-11.0×10³/µL
Hemoglobin	13.0 g/dL	Female: 12.0-15.5 g/dL
Hematocrit	41%	34.9 - 45.2 %
Mean corpuscular volume	96.5 fL	79.3 - 98.6 fL
Platelet count	292 ×10³/µL	150-400×10³/µL
Metabolic panel		
Sodium	142 mmol/L	135 - 146 mmol/L
Potassium	4.3 mmol/L	3.6 - 5.3 mmol/L
Chloride	105 mmol/L	96 - 106 mmol/L
Bicarbonate	28 mmol/L	20 - 30 mmol/L
Creatinine	0.45 mg/dL	0.60 - 1.30 mg/dL
Glucose	95 mg/dL	65 - 99 mg/dL

**Table 2:** Cerebrospinal fluid analysis IgG: Immunoglobulin G

Test	Value	Reference range
Cerebrospinal fluid		
Red blood cell count	1 /cmm	0 - 10 /cmm
White blood cell count	3 /cmm	0 - 5 /cmm
Lymphocyte	87%	40 - 80 %
Protein	55 mg/dL	15 - 45 mg/dL
Glucose	62 mg/dL	43 - 73 mg/dL
Neuronal Nuclear Antibodies IgG ImmunoBlot	Ri detected	None detected
Neuronal Nuclear Antibody Titer, IgG	01:05	<1:1

She was initiated on intravenous immunoglobulin (IVIG), intravenous corticosteroids, and rituximab, and underwent urgent right mastectomy and lymph node dissection. Hormone therapy with an aromatase inhibitor was also initiated. Despite these interventions, her neurologic symptoms progressed, resulting in respiratory failure requiring endotracheal intubation and subsequent tracheostomy placement. She ultimately opted to transition to comfort-focused care and was discharged from the hospital with hospice services.

## Discussion

This report describes a case of PNS that occurred in a patient with an untreated breast malignancy. PNS are challenging to diagnose due to the wide range of clinical presentations and syndromes, low incidence, and nonspecific imaging findings. Antibody testing can be helpful in the diagnosis, although certain antibodies (classified as “high risk”) are highly associated with malignancy, whereas others demonstrate a weak association [4].

In our case, CSF antibody testing demonstrated positive anti-Ri antibodies. In women in whom a diagnosis of malignancy has not been made, the detection of anti-Ri antibodies is considered “high risk” for the presence of breast cancer, with an association of greater than 70%; associated neurologic disorders include cerebellar degeneration and opsoclonus-myoclonus-ataxia syndrome (OMAS) [3-5]. In these disorders, patients may present with progressive ataxia, eye movement disorders, tremors, myoclonus, and dysarthria. Studies suggest that Nova-1, a protein found both in tumors as well as neurons, is the target antigen of anti-Ri antibodies; however, the mechanism through which this results in the clinical phenotypes described above is poorly understood [6]. Pathology findings from patients with OMAS typically demonstrate perivascular and interstitial inflammatory infiltrates involving the brainstem, cerebellum, and leptomeninges, and Purkinje cell loss [3,7]. 

Aside from antibody testing in the serum and CSF, other diagnostic modalities, including neuroimaging and routine laboratory evaluation, are typically unremarkable or nonspecific in PNS. For instance, in one series of patients with OMAS, the majority of patients had normal neuroimaging [8]. CSF evaluation may demonstrate mild elevations of protein or mild lymphocytic pleocytosis in certain patients [9].

The mainstay of PNS treatment remains treatment of the underlying malignancy. Although immunomodulatory therapy may be beneficial in certain types of PNS, including myasthenia gravis (associated with thymoma) and Lambert-Eaton syndrome (associated with small cell lung cancer), its role in anti-Ri-associated PNS is less clear [10]. In a series of 10 patients with paraneoplastic OMAS, neurologic symptoms progressed in all four patients who did not receive antineoplastic therapy, despite aggressive immunomodulatory therapies; in contrast, all patients who received antineoplastic therapy experienced clinical improvement [8]. However, given the ongoing ectopic expression of ectopic tumor antigens and persistent immune response noted in certain cases of PNS, immunomodulatory therapy is often used in conjunction with or after antineoplastic therapy [11]. In our case, the patient underwent initial surgical resection of the tumor but was lost to medical follow-up and did not receive adjuvant systemic antineoplastic therapy, ultimately developing progression of her PNS symptoms.

## Conclusions

PNS represent a diverse range of clinical syndromes with varying degrees of association with malignancies. Due to their heterogeneity, PNS are challenging to diagnose; traditional diagnostic modalities, including neuroimaging and routine serum and CSF laboratory evaluation, may be normal or demonstrate nonspecific results. Antibody testing may be helpful, but its specificity and association with specific malignancies vary depending on the specific antibody. Although immunomodulatory therapies may be beneficial for some patients, the treatment of PNS typically requires treatment of the underlying malignancy.
